# Diagnostic Capacity for Fungal Infections in Tertiary Hospitals in Nigeria and Ghana - An Onsite Baseline Audit of 9 Sites

**DOI:** 10.3389/ijph.2024.1607731

**Published:** 2024-11-14

**Authors:** Damilola Akinlawon, Iriagbonse Osaigbovo, Mohammed Yahaya, Olufunmilola Makanjuola, Ubong A. Udoh, Philip Nwajiobi-Princewill, Ifeyinwa Nwafia, Jonah Peter, Isabella Asamoah, Folake Peters, Obiora Okafor, Tochi Okwor, Akin Osibogun, Folashade Ogunsola, Alexander Jordan, Tom Chiller, Rita Oladele

**Affiliations:** ^1^ Department of Community Health and Primary Care, Lagos University Teaching Hospital, Lagos, Nigeria; ^2^ Department of Medical Microbiology, College of Medical Sciences, University of Benin, Benin, Nigeria; ^3^ Department of Medical Microbiology and Parasitology, College of Medical Sciences, Usmanu Danfodiyo University Teaching Hospital, Sokoto, Nigeria; ^4^ Department of Medical Microbiology and Parasitology, University of Ibadan, Ibadan, Nigeria; ^5^ Department of Medical Microbiology and Parasitology, University of Calabar, Calabar, Nigeria; ^6^ Department of Medical Microbiology and Parasitology, National Hospital, Abuja, Nigeria; ^7^ Department of Medical Microbiology and Parasitology, University of Nigeria Teaching Hospital, Enugu, Nigeria; ^8^ Department of Medical Microbiology and Parasitology, University of Abuja Teaching Hospital, Abuja, Nigeria; ^9^ Department of Infectious Diseases, Korle Bu Teaching Hospital, Accra, Ghana; ^10^ Department of Medical Microbiology and Parasitology, Lagos University Teaching Hospital, Lagos, Nigeria; ^11^ Nigeria Centre for Disease Control, Abuja, Nigeria; ^12^ Department of Community Health and Primary Care, College of Medicine University of Lagos, Lagos, Nigeria; ^13^ Department of Medical Microbiology and Parasitology, College of Medicine, University of Lagos, Lagos, Nigeria; ^14^ Mycotic Diseases Branch, Division of Foodborne, Waterborne, and Environmental Disease, Centers for Disease Control and Prevention, Atlanta, GA, United States

**Keywords:** laboratory audit, fungal infections, diagnosis, resource limited setting, tertiary hospital

## Abstract

**Objectives:**

To assess diagnostic mycology capacity and available fungal diagnostic services of microbiology laboratories in eight tertiary hospitals in Nigeria and one in Ghana.

**Methods:**

On-site audits were performed in the microbiology laboratories of nine tertiary hospitals using a structured observation checklist.

**Results:**

A total of nine tertiary hospitals' laboratories in Nigeria and Ghana were assessed between June 2022 and December 2023. The majority of audited laboratories lacked basic infrastructure and materials needed for fungal diagnostic testing, with less than half of the labs having a dedicated mycology bench, space or room, 3/9 (33.3%), appropriate bench workflow 1/9 (11.1%), functional biosafety cabinet type two 2/9 (22.2%), dedicated incubators 3/9 (33.3%), standard operating procedures 1/9 (11.1%), mycology atlases 2/9 (22.2%). Trained laboratory personnel for mycology were also lacking with only one of the laboratories 1/9 (11.1%) observed to have a designated trained personnel for the mycology bench.

**Conclusion:**

The audit revealed deficits in basic infrastructure, material resources, dedicated human resources, and laboratory capacity to detect serious fungal infections.

## Introduction

Over a billion people are estimated to have fungal infections, 15%–30% of which are serious [1]. Globally, millions are estimated to die from serious fungal infections annually (such as cryptococcal meningitis, invasive candidiasis, invasive aspergillosis and mucormycosis), yet these diseases have received little public health attention [2]. The World Health Organization recently made concerted efforts to tackle this concern and produced a fungal priority pathogens list, in order to enhance effective global responses towards their amelioration [3]. A substantial proportion of deaths due to fungal diseases are preventable if detected early and with fast initiation of appropriate therapy [4]. However, the symptoms of serious fungal diseases are often non-specific and their diagnosis relies on a high index of clinical suspicion supported by imaging and laboratory investigations., [5] Access to appropriate laboratory processes and diagnostic methodologies are vital to ensure accurate and rapid detection of fungal pathogens.

Many resource-limited countries currently struggle with weak laboratory systems which lack adequate infrastructure and human resources [6]. Attempts at laboratory systems strengthening typically adopt a vertical approach targeting diseases of recognized public health importance such as HIV/AIDS, tuberculosis, malaria, multi-drug resistant bacteria and most recently COVID-19. Due to a historical lack of awareness and funding for fungal disease initiatives, it is unsurprising that recent surveys of fungal diagnostic capacity in Africa, including Nigeria, report critical gaps even at the tertiary laboratory level [6–8]. Moreover, diagnosing fungal infections is uniquely challenging due to the expertise required to accurately identify many fungal pathogens, a limited number of available diagnostic modalities for fungal identification (compared to that of bacteria and viruses), and more limited access to these modalities in many parts of the world [9]. Although there have been many recent advances in laboratory technology for the biological identification and characterization of serious fungal diseases (e.g., molecular assays) [10, 11], most of these novel diagnostics are yet to become available in low resource settings.

The exact burden of serious fungal infections in Nigeria and Ghana remains uncertain though it has been estimated that 11.8% of Nigerians and 4% of Ghanaians are affected annually [12, 13]. Disease surveillance is a critical tool to determine the burden of the problem and also to track emergence of resistance. Fungal diseases surveillance was initiated as a program in Nigeria and Ghana in 2022 and 2024, respectively. Nine tertiary hospitals were selected as sentinel sites to implement the program’s initiatives. An audit of diagnostic mycology services available in the microbiology laboratories of these tertiary hospitals was conducted to provide a baseline for the monitoring and evaluation of capacity building efforts at the laboratories. The audit findings are presented in this manuscript.

## Methods

This audit was in two phases;

A. A laboratory audit (infrastructure, personnel and processes), which was conducted by three persons (the project manager, site investigator and principal investigator).
B. A review of the mycology registers by an identified dedicated focal laboratory personnel (an individual laboratory scientist nominated by the head of the laboratory).

A. This laboratory audit was a physical on-site audit performed in nine tertiary hospitals: eight in Nigeria and one in Ghana. A clinical audit cycle methodology consisting of five steps (preparing for the audit, setting criteria and parameters to be measured data collection, data analysis and implementation of changes, and improvements checking and maintenance [14]) was deployed.

Hospital management at the sites were informed and gave permission for the audit prior to the audit teams arrival at the respective facilities.

Data on the infrastructural capacity of sites to provide diagnostic services for mycology was collected using a checklist containing the adopted parameters. Each item on the checklist was recorded as present or absent after inspection, during the on-site audits. The duration of this audit was 3 days in each of the sites.

The following parameters were selected as standard for assessment. These parameters were adopted and adapted from the European Confederation of Medical Mycology (ECMM) Excellence Centers Quality Audit policy document (Blue status centers – minimum requirements) [14]:

i. Presence of a dedicated mycology laboratory room/space/bench
ii. Workflow of the bench (with separation of areas to limit contamination)
iii. Dedicated staff for the bench/lab/room
iv. Presence of a functional biosafety cabinet (type 2 minimum, to protect the laboratory personnel from aerosols)
v. Existing functional microscopes for the bench/room/space
vi. Dedicated incubator
vii. Fridges and freezers (preferably dedicated to the mycology work, this is for storage, important because molds generate spores that can lead to cross-contamination)
viii. Presence (visualized) of Standard Operating Procedures for processing mycology specimens.
ix. Mycology atlases (for identification of isolates/pathogens)
x. Dedicated Laboratory register(s) for mycology bench/room work
xi. Number and type of specimen requests for mycology processing and fungal pathogens isolated.
xii. Vitek 2 machine (for fungal identification and antifungal susceptibility testing)
xiii. Cryptococcal antigen test (CrAg)
xiv. Histoplasma ELISA test
xv. Electricity supply

B. Laboratory mycology data collation: The dedicated focal laboratory personnel collated retrospective data on fungal specimen testing requests and on fungal pathogens identified in each laboratory (all benches) for the preceding 2 years. Specimen for fungal studies documented as “sent,” included specimen from either superficial, subcutaneous or invasive sites. While fungal isolates captured in this report, were collated from the mycology register and from the results from the “sterile bench” (blood culture, cerebrospinal fluid). Data collation duration was 1 month.

## Results

**FIGURE 1:**
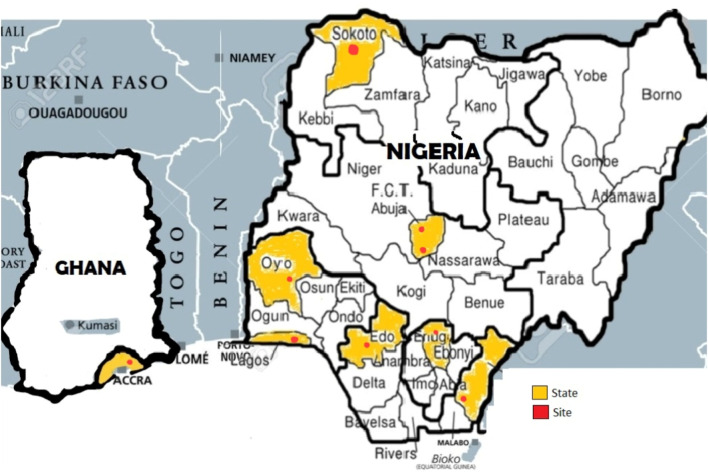
Distribution of sites in Nigeria and Ghana (Nigeria, Ghana, 2024).

A total of nine medical microbiology laboratories located in tertiary hospitals in Nigeria and Ghana were assessed between June 2022 and December 2023. Figure 1 shows a distribution of the sites. A third of the laboratories, 3/9 (33.3%) had a dedicated mycology bench/space/room, and only one of the laboratories 1/9 (11.1) had a designated and trained personnel for the mycology bench. One of the laboratories, 1/9 (11.1%) had the ideal bench workflow. Two of the laboratories, 2/9 (22.2%) had a functional biosafety cabinet type 2. The biosafety cabinets in two other facilities, though present, were either past due for inspection or had failed inspection. More than half of the laboratories audited, 5/9 (55.6%) had dedicated functional microscopes, and fridges and freezers dedicated for mycology. Three (33.3%) of the laboratories assessed, had dedicated incubators for mycology. One (11.1%) of the sites had existing standard operating procedures for the laboratory diagnosis of various fungal pathogens, while 2/9 (22.2%) had mycology atlases sighted (Table 1).

**TABLE 1:** Laboratory audit summary (Nigeria, Ghana, 2024).

Parameters	Site 1	Site 2	Site 3	Site 4	Site 5	Site 6	Site 7	Site 8	Site 9
Presence of a dedicated mycology laboratory room/space/bench	Yes	No	No	Yes	No	No	No	Yes	No
Dedicated personnel for the bench	Yes	No	No	No^a^	No	No	No	No	No
Workflow of the bench (with separation of areas to limit contamination)	Yes	No	No	No	No	No	No	No	No
Functional biosafety cabinet (type 2 minimum)	No^b^	Yes	No	No	No	No^c^	No	Yes	No
Dedicated functional microscopes	Yes	Yes	Yes	Yes	No	Yes	No	No	No
Dedicated incubator	Yes	No	Yes	Yes	No	No	No	No	No
Fridges and freezers (preferably dedicated to the mycology work	Yes	Yes	Yes	Yes	No	Yes	No	No	No
SOPs	Yes	No	No	No	No	No	No	No	No
Mycology atlases	Yes	Yes	No	No	No	No	No	No	No
Laboratory registers	Yes	Yes	Yes	Yes	Yes	Yes	Yes	Yes	Yes
Electricity supply (per 24 h)	20–24 h	12–20 h	12–20 h	8–12 h	12–20 h	20–24 h	12–20 h	20–24 h	20–24 h
Vitek 2 machine	Yes	Yes	Yes	Yes	Yes	Yes	Yes	Yes	No
CrAg test	Yes	Yes	No	No	No	No	No	Yes	Yes
Histoplasma ELISA	Yes	No	No	No	Yes	No	No	No	No
Others – Bunsen burners, paraffin wax/tape for sealing plates, india ink, yeast cards, cryotubes, cryoracks, lactophenol blue	Yes	No	No	No	No	No	No	No	No

^a^ Trained staff available, but due to low workload was seconded to work elsewhere.

^b^ Present but failed inspection.

^c^ Present but due for inspection.

All sites audited had dedicated laboratory registers for fungal studies, however the review revealed a dearth (0–208) of samples sent for fungal studies and few (0–94) fungal isolates identified annually (see Table 2). The laboratory record for the Ghanaian site also did not have mycology entries on mycology samples and isolates identified, however CrAg test results was being done for the HIV program. In the audit, it was ensured that all fungi (yeast and molds) identified in the routine microbiological investigations for the sites was captured in the data collation. Electricity supply was constant (20–24 h supply daily) in less than half of the laboratories 4/9 (44.4%), and the others had poor power supply which was either erratic (12–20 h supply daily) 4/9 (44.4%) or epileptic (8–12 h supply daily) 1/9 (11.1%). Most of the sites, 8/9 (88.9%) possessed a Vitek-2 machine, however, as of the time of the audit only one site 1/9 (11.1%) had a sustainable system to acquire yeast cards to be operated with the machine. Less than half of the laboratories had the CrAg test 4/9 (44.4%) and only 2/9 (22.2%) had an ELISA reader to conduct the Histoplasma immunoassays.

**TABLE 2:** Retrospective mycology laboratory record review 2020/2021 (Nigeria, Ghana, 2024).

	Site 1	Site 2	Site 3	Site 4	Site 5	Site 6	Site 7	Site 8	Site 9
Number of beds	900	910	550	1,400	410	850	500	350	2,000
Number of mycology samples 2020 (2022)	126	52	42	38	66	76	16	83	0 (No mycology bench/lab register)
Number of fungal isolates 2020 (2022)	23	11	14	10	62	32	14	78	-None
Number of mycology samples 2021	208	38	65	28	72	118	21	66	0 (No mycology bench/lab register)
Number of fungal solates 2021	94	10	27	12	65	49	7	65	-None
Isolates Documented)	*Candida* spp. *Aspergillus* spp. *Microsporum* spp. *Cryptococcus* spp. *Fusarium* spp. *Cladosporum* spp. *Trichophyton* spp.	*Aspergillus* spp. *Candida* spp. *Cryptococcus neoformans* *Penicillium* spp.	*Aspergillus* spp. *Candida* spp. *Mucor* spp. *Penicillium* spp.	*Candida* spp. *Aspergillus* spp. *Fusarium* spp. *Trichophyton* spp.	*Candida* spp. *Aspergillus* spp. *Trichophyton* spp.	*Aspergillus* spp. *Candida* spp. *Cryptococcus laurentii*	*Candida* spp. *Trichophyton* spp.	*Candida* spp. *Trichophyton* spp. *Aspergillus* spp. *Cryptococcus* spp.	-None

## Discussion

Laboratory audits objectively measure laboratory infrastructure, personnel, and practices against valid and explicit standards in order to identify and implement appropriate change [15]. While assessments of this nature may be challenging in resource limited settings, they are essential for stimulating quality improvement and strengthening laboratory systems [15]. As fungi have been historically neglected in clinical and laboratory contexts globally, and in Africa especially, it was deemed necessary to perform an audit of the laboratories in selected sites prior to implementing capacity building and fungal diseases surveillance in our setting. This audit revealed significant structural, diagnostics, capacity and process deficits for fungal studies in most of the laboratories assessed. This finding is expected given that mycology tends to be less prioritized than bacterial and viral microbiology in many clinical settings across Africa [7].

Our findings differ from an Asian report which revealed a much higher proportion of laboratories with dedicated infrastructure (53.5%), personnel and diagnostics for mycology [16]. They also reported a much higher number of fungal samples weekly. Majority (127/238,53.4%) of the laboratories received 0–50 mycology samples weekly and only 9.7% of laboratories (23/238) received more than 500 samples weekly [16]. It is important to note that these were bigger facilities. In a recent audit report from Australia, they found significant variation in practices, many of which fell short of recommended procedures from an earlier audit [17].

The ECMM Excellence Centers Quality Audit policy utilized in this study ensures laboratories meet internationally recognized standards for mycology services. “Blue status centers” represent the minimum requirements for infrastructure, personnel, and diagnostics promoting standardization, performance evaluation, continuous improvement, and laboratory quality compliance in contributing to better diagnosis and management of fungal infections [18]. In this audit, only one of the laboratories (11.1%) potentially fulfilled the minimum laboratory requirements for ECMM Excellence Centre blue status. Similarly, a survey carried out by the ECMM to assess the state of clinical mycology in Africa by focusing on laboratory infrastructure and antifungal availability revealed only five institutions (12·5%) of 40 located in Cameroon, Kenya, Nigeria, Sudan, and Uganda potentially fulfilled the minimum laboratory requirements [18].

There have been a few recent assessments of laboratory capacity for diagnostic mycology in Nigerian laboratories but this audit differs from others in methodology and purpose [6–8]. Unlike others which employed on-line surveys and key informant interviews to obtain data, this was a physical audit that enabled the auditors to garner nuanced information including the actual functionality of diagnostic and lab equipment. Also, while other studies aimed primarily to inform and advocate for better investments in fungal diagnostics from governments and policymakers, the present audit was targeted at identifying areas for near-term intervention through provision of diagnostic mycology services and training for quality improvement purposes [6–8].

Interventions which fall within the plan of the fungal diseases’ surveillance program include training for personnel in each of the laboratories on culture-based and non-culture-based diagnostics for fungi, and provision of mycology atlases and SOP development for use in the various laboratories. However other provisions did not fall within the remit of the program, such as the procurement of equipment such as biosafety cabinets, incubators and ELISA readers and provision of dedicated work benches for mycology were captured in recommendations to laboratory leadership and facility heads with advocacy to invest more in diagnostic mycology. It was also recommended that laboratory managers of the audited sites find creative ways to leverage on existing equipment not domiciled in the microbiology laboratory but present elsewhere in the facility such as ELISA readers in chemistry or biosafety cabinets in dedicated TB laboratories. A key observation of the audits was the lack of trained personnel designated to process mycology samples in most of the laboratories. Conventional laboratory diagnosis of fungal diseases requires personnel highly-skilled in specimen processing, isolation and identification [9]. Even in developed countries, this expertise has been on a consistent decline among routine clinical microbiology laboratory personnel [9]. This is largely because medical mycology is often underemphasized in the education and training of medical and other health professionals [9, 19]. In these developed countries, the uptake of newer automated techniques and molecular methodologies partially offsets the loss of skill in traditional mycology methods. Since access to these newer technologies is often limited in lower resource settings, the ability to identify key fungal pathogens based on morphologic and phenotypic characteristics must continue to be a priority.

Additionally, relatively few fungal pathogens besides *Candida spp*. and *Aspergillus* spp. have been targeted with the new technologies, such as the histoplasma antigen and cryptococcal antigen tests so there is a continued need for personnel skilled in traditional methods to identify the other fungi capable of causing disease in humans, and especially in immunocompromised populations. The audit also uncovered a dearth of specimens received for diagnostic mycology which could be due to a lack of formally trained laboratory personnel as well as other reasons such as low index of suspicion amongst clinicians. Based on these audit findings, the training of personnel at the various sites was bookmarked as a major area for intervention by the fungal diseases surveillance and capacity building program. The highest scoring laboratory was selected to provide peer mentorship for the other laboratories, as well as to serve as a site for intensive training of laboratory personnel from the other sites.

The countries’ national plans to combat antimicrobial resistance incorporate surveillance for multi-drug resistant bacteria requiring accurate organism identification and susceptibility testing [20]. Hence it was not surprising that all the laboratories, many of which participate in the national AMR surveillance network, had the Vitek-2 machine, which provides automated identification and susceptibility testing. However, because the emphasis has been on multi-drug resistant bacteria, and not fungi, yeast identification cards and antifungal susceptibility testing cards were not supported in all of the laboratories. One of the main limitations of this audit was that only one site in Ghana was included, however, it is worthy to note that this is the biggest tertiary facility in Ghana. Thus, one can deduct that other tertiary centers will have even less capacity. This is not surprising because at the on-site audit it was observed that most laboratory samples were routinely sent to private/external laboratories, and this appeared to be the practice state-wide. Another limitation was the small sample size (just nine sites), however, this was because these were the tertiary hospital sites selected for fungal disease surveillance.

The fungal diseases surveillance program is providing fungal disease diagnostics such as the cryptococcal antigen lateral flow assay test, *Aspergillus* galactomannan, *Aspergillus* IgG, and *Histoplasma* enzyme immunoassay which are not routine tests in most of the laboratories. The program also built capacity for laboratory diagnosis and management of fungal infections. Advocacy using data from the surveillance program will be made to the Nigerian and Ghanaian governments through their Federal Ministry of Health, and other national public health bodies (e.g.,: Nigeria Centre for Disease Control for Nigeria). The advocacy will be to consider fungal diseases as a public health concern and the need to sustain the supply of these diagnostics beyond the program’s activities. To complete the audit cycle and to evaluate the success of the program interventions, future evaluations need to be scheduled using the same data collection strategy as employed in the initial audit for comparability. The ultimate aim is to enable continuous quality improvement in fungal disease diagnosis in each of the selected laboratories.

### Conclusion

Our findings revealed a considerable lack of infrastructure, diagnostics and dedicated human resources required for the laboratory detection of serious fungal infections. While non-governmental initiatives such as the ongoing fungal diseases surveillance program may bridge some of these gaps, advocacy to pertinent healthcare stakeholders is critical to sustain the gains from the fungal diseases surveillance and capacity building program. Going forward, routine audits of this nature may be considered as a means to continually assess and build capacity for the detection of neglected fungal diseases in resource limited settings.
